# Neoadjuvant immunotherapy for resectable esophagal squamous cell carcinoma: An ace up the sleeve

**DOI:** 10.1002/ctm2.1426

**Published:** 2023-09-21

**Authors:** Qing Zhou, Jingnan Yuan, Kui Wu, Jun Yin, Lijie Tan

**Affiliations:** ^1^ Guangdong Provincial Key Laboratory of Human Disease Genomics Shenzhen Key Laboratory of Genomics, BGI Research Shenzhen China; ^2^ HIM‐BGI Joint Lab, Hangzhou Institute of Medicine (HIM) Chinese Academy of Sciences, BGI Research Hangzhou China; ^3^ Zhejiang Cancer Hospital, Hangzhou Institute of Medicine (HIM) Chinese Academy of Sciences Hangzhou China; ^4^ Department of Thoracic Surgery Cancer Center Zhongshan Hospital of Fudan University Shanghai China; ^5^ Department of Thoracic Surgery, Zhongshan Hospital (Xiamen) Fudan University Xiamen China

**Keywords:** biomarkers, locally advanced oesophagal squamous cell carcinoma, neoadjuvant immunotherapy

## 1 MAIN TEXT

The emergence of immune checkpoint agents has transformed neoadjuvant therapy into a life‐saving treatment with curative promise. Mounting evidence in neoadjuvant immunotherapy has led to improvement in pathological tumour regression in patients with resectable oesophagal cancer. The latest results of the phase 1b trial (NATION‐1907) in the issue of *Nature Medicine*, suggest that neoadjuvant anti‐programmed death ligand 1 (anti‐PD‐L1) alone may induce significantly prolonged survival outcomes in resectable oesophagal squamous cell carcinoma (ESCC).

## 2 NEOADJUVANT ANTI‐PD‐L1 BLOCKADE EFFECTIVELY AGAINST DISEASE RELAPSE

Distant and locoregional relapses of locally advanced ESCC remain at 48% after standard‐of‐care neoadjuvant chemotherapy (nCT) or chemoradiotherapy (nCRT) followed by surgery.^1^ To explore new therapeutic strategies to reduce relapse and prolong overall survival (OS) is still an unmet need. Immune checkpoint blockade has revolutionized the treatment paradigm and is gradually administrated in patients with locally advanced ESCCs in the postoperative adjuvant and preoperative neoadjuvant setting. Two key studies have demonstrated that immunotherapy has remarkably delayed disease relapses. That is, adjuvant nivolumab has significantly improved disease‐free survival in the Checkmate‐577 trial^2^ while neoadjuvant adebrelimab (anti‐PD‐L1) blockade has substantially prolonged 2‐year recurrence‐free survival (100%) and 2‐year OS (92%) in the NATION‐1907 trial.^3^

Preliminary evidence highlight three clinical opinions on the clinical efficacy and safety of neoadjuvant therapy in patients with locally advanced resectable ESCC, that is, 1) neoadjuvant anti‐PD‐L1 blockade alone followed surgery have superior OS compared with standard‐of‐care treatments; Post doc analysis demonstrated that the 2‐year OS was 94% in neoadjuvant anti‐PD‐L1 therapy, compared with 69% with nCRT (hazard ratio [HR] .17, 95% confidence interval [95% CI] .04–.76; *p* = .021) and 67% with nCT (HR .13, 95% CI .03–.59; *p* = .008); 2) neoadjuvant anti‐PD‐L1 blockade alone have non‐inferior OS compared with neoadjuvant chemoimmunotherapy followed surgery in some small phase II trials; Analysis of propensity score adjustment in patient baseline characteristics showed that the 2‐year OS was 93% in neoadjuvant anti‐PD‐L1 therapy, compared with 74%–87% with neoadjuvant anti‐PD‐1 blockade plus chemotherapy (*p* = .066); 3) neoadjuvant anti‐PD‐L1 blockade have significantly favorable safety profiles compared with standard‐of‐care or chemoimmunotherapy, which are friendly for patients over 70 years old or intolerant of chemotherapy or chemoradiotherapy. As a caveat, these conclusions are based on post‐doc comparative analysis after propensity score matching and should be further validated in randomized, controlled phase III trials.

These benefits of neoadjuvant immunotherapy have been interpreted to be associated with the enhanced systemic anti‐tumour effects and reinvigorated responsive T cells against tumour antigens upon blockade of the PD‐1/PD‐L1 axis, eliminating micro‐metastatic tumour cells which may lead to postsurgical relapse.^4^ Whereas nCT or nCRT can “debulk” tumours preoperatively, neoadjuvant immune checkpoint blockade aims to enhance systemic antitumor immunity based on the rationale of promoting greater tumour‐reactive T cell expansion of pre‐existing intratumoral clones at baseline and heighten systemic micro‐metastases surveillance. Given the complete surgical resection and radical lymphadenectomy are fulfilled, neoadjuvant therapy should be more focused on systemic antitumor effects rather than locoregional tumour control.

## 3 PCR MAY NOT BE A PERFECT SURROGATE FOR OS IN RESECTABLE ESCC

Traditionally, patients with pathological complete response (pCR) are expected to have longer OS than non‐pCR patients in resectable ESCCs. However, achievement of non‐pCR does not represent short‐term outcomes. Recent findings of two studies provide evidence challenging the validity of local tumour regression (pCR rate) as a surrogate for OS in the neoadjuvant setting. Although combined therapeutic strategies contribute to substantial improvement of pCR rates, they might not translate into OS benefits. This is based on the notion that there is no statistical difference in OS between nCRT and nCT in patients with stage cT3‐4aN0‐1M0 in the randomized, controlled phase III CMISG‐1701 trial (3‐year OS, 64.1% versus 54.9%, *p* = .28; pCR, 27.7% versus 2.9%, *p* < .001),^5^ as well as immunotherapy alone and chemoimmunotherapy in patients with stage cT2‐4aN0‐2M0 in the NATION‐1907 trial (2‐year OS, 93% versus 74%−87%, *p* = .066; pCR, 8.0% versus 29.0%, *p* < .001).^3^ It is speculated that better local tumour control fails to translate into survival benefits attributed to combination regimens compromising the clinical efficacy of immunotherapy through impairing the clonal expansion of intratumorally pre‐existing tumour‐reactive T cells specific for mutation‐associated neoantigens.^6^, ^7^ Taken together, evaluation of OS (or recurrence‐free survival, progression‐free survival) should be the key determinant to decide whether a therapeutic regimen brings long‐term survival, instead of pathological response(pCR).

## 4 PERSONALIZED AND BIOMARKER‐DRIVEN NEOADJUVANT IMMUNE CHECKPOINT BLOCKADE

How to choose an appropriate neoadjuvant approach in combination with immunotherapy in resectable ESCC remains controversial. Preliminary results recently reported that a 2‐year OS of neoadjuvant single‐agent anti‐PD‐L1 was slightly superior to anti‐PD‐1 plus chemotherapy, both of which prolonged survival outcomes compared with nCT or nCRT, suggesting that biomarker‐driven patient selection may be crucial for de‐escalating chemotherapy and radiotherapy when neoadjuvant immunotherapy is given in resectable ESCC.

**FIGURE 1:**
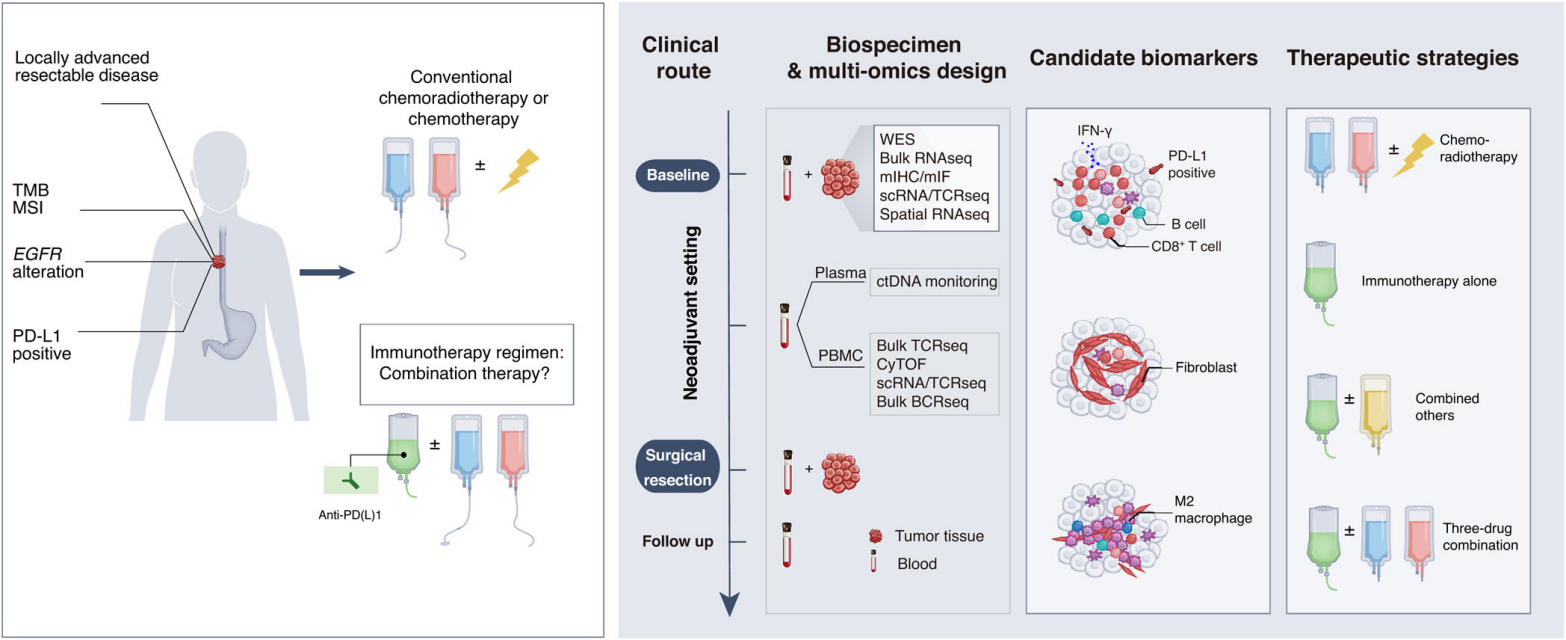
Precision neoadjuvant therapeutics strategies for locally advanced resectable ESCC. Many factors may have impacts on the clinical efficacy of immunotherapy with or without other treatments (namely, chemotherapy or chemoradiotherapy). Biomarker‐driven neoadjuvant treatment regimens may be explored based on multi‐omics exploratory analysis in the early‐phase studies when longitudinal peripheral blood and tumour specimens are available. Optimal clinical scenarios of two‐drug or three‐drug combination (anti‐PD‐(L)1 and chemotherapy) should be carefully determined in clinical practice in case of overtreatment and increasing treatment‐related adverse events. TMB, Tumor mutational burden; MSI, microsatellite instability; EGFR, epidermal growth factor receptor; PD‐L1, programmed death ligand 1; WES, whole‐exome sequencing; mIHC, multiplex immunohistochemistry; mIF, multiplex immunofluorescence; TCR, T cell receptor; BCR, B cell receptor; CyTOF, cytometry by Time‐Of‐Flight; scRNA, single‐cell RNA sequencing; IFN‐γ, Interferon γ.

Typical biomarkers, such as PD‐L1 expression, microsatellite instability, and tumour mutation burden, have been demonstrated inconsistent in predicting therapeutic response in the neoadjuvant setting. Therefore, interest has turned to focus on the composition of the tumour microenvironment in resected tumours and peripheral blood, which may illustrate sensitive or resistant mechanisms and suggest future biomarkers. The NATION‐1907 trial has identified ‘Interferon/Epithelial‐mesenchymal transition (IFN/EMT) score’ signature and immune‐enriched tumour microenvironment phenotype to stratify for well responders who would benefit from neoadjuvant adebrelimab blockade, highlighting that anti‐PD‐L1 monotherapy may be sufficient in the immune “inflamed” patients. For patients with high infiltration of fibroblasts at baseline, immunotherapy combined with other drugs to shift the fibrotic tumour microenvironment may be a beneficial therapeutic strategy. PD‐L1 positive (combined positive score ≥10) shows superior clinical survival regardless of single‐agent immunotherapy or combined with other regimens.^3^, ^8^ Clinical trials about the head‐to‐head comparison of clinical efficacy of different neoadjuvant regimens and investigating the utility of these candidate biomarkers would identify the optimal therapeutic regimen and should be further investigated in expanded cohorts (Figure 1).

## 5 PERSPECTIVE

Despite a flurry of encouraging discoveries and clinical benefits emerging from neoadjuvant immunotherapy with or without combinations of other agents, coupled with that translational research is still only scratching the surface of possibilities. The NATION‐1907 trial has confirmed a non‐inferior OS and favourable safety profiles of neoadjuvant immunotherapy compared to standard‐of‐care treatments. Whether the synergistic effects of immunotherapy and chemotherapy or chemoradiotherapy can overpower the side effects and bring survival benefits remains to be further investigated. With an elaborate design, impactful correlative studies can be fulfilled in a rather small‐sized clinical trial, which might eventually convey details surrounding the mechanism of response or resistance to immunotherapy.

## CONFLICT OF INTEREST STATEMENT

The authors declare no conflict of interest.

## FUNDING INFORMATION

This work was supported by the Guangdong Provincial Key Laboratory of Human Disease Genomics (2020B1212070028), The Science and Technology Commission of Shanghai Municipality (20ZR1411600), National Natural Science Foundation of China (82241013), Shanghai Hospital Development Center (SHDC2020CR4039).
